# Human resources for health: task shifting to promote basic health service delivery among internally displaced people in ethnic health program service areas in eastern Burma/Myanmar

**DOI:** 10.3402/gha.v7.24937

**Published:** 2014-09-29

**Authors:** Sharon Low, Kyaw Thura Tun, Naw Pue Pue Mhote, Saw Nay Htoo, Cynthia Maung, Saw Win Kyaw, Saw Eh Kalu Shwe Oo, Nicola Suyin Pocock

**Affiliations:** 1 Community Partners International, Mae Sot, Thailand; 2 Burma Medical Association, Mae Sot, Thailand; 3 Health Information System Working Group, Mae Sot, Thailand; 4 Back Pack Health Worker Team (BPHWT), Mae Sot, Thailand; 5 Mae Tao Clinic, Mae Sot, Thailand; 6 Karen Department of Health and Welfare (KDHW), Mae Sot, Thailand; 7 Department of Global Health and Development, London School of Hygiene and Tropical Medicine, London, UK

**Keywords:** health system strengthening, health workforce, task shifting, internally displaced people, Burma/Myanmar

## Abstract

**Background:**

Burma/Myanmar was controlled by a military regime for over 50 years. Many basic social and protection services have been neglected, specifically in the ethnic areas. Development in these areas was led by the ethnic non-state actors to ensure care and the availability of health services for the communities living in the border ethnic-controlled areas. Political changes in Burma/Myanmar have been ongoing since the end of 2010. Given the ethnic diversity of Burma/Myanmar, many challenges in ensuring health service coverage among all ethnic groups lie ahead.

**Methods:**

A case study method was used to document how existing human resources for health (HRH) reach the vulnerable population in the ethnic health organizations’ (EHOs) and community-based organizations’ (CBHOs) service areas, and their related information on training and services delivered. Mixed methods were used. Survey data on HRH, service provision, and training were collected from clinic-in-charges in 110 clinics in 14 Karen/Kayin townships through a rapid-mapping exercise. We also reviewed 7 organizational and policy documents and conducted 10 interviews and discussions with clinic-in-charges.

**Findings:**

Despite the lack of skilled medical professionals, the EHOs and CBHOs have been serving the population along the border through task shifting to less specialized health workers. Clinics and mobile teams work in partnership, focusing on primary care with some aspects of secondary care. The rapid-mapping exercise showed that the aggregate HRH density in Karen/Kayin state is 2.8 per 1,000 population. Every mobile team has 1.8 health workers per 1,000 population, whereas each clinic has between 2.5 and 3.9 health workers per 1,000 population. By reorganizing and training the workforce with a rigorous and up-to-date curriculum, EHOs and CBHOs present a viable solution for improving health service coverage to the underserved population.

**Conclusion:**

Despite the chronic conflict in Burma/Myanmar, this report provides evidence of the substantive system of health care provision and access in the Karen/Kayin State over the past 20 years. It underscores the climate of vulnerability of the EHOs and CBHOs due to lack of regional and international understanding of the political complexities in Burma/Myanmar. As Association of Southeast Asian Nations (ASEAN) integration gathers pace, this case study highlights potential issues relating to migration and health access. The case also documents the challenge of integrating indigenous and/or cross-border health systems, with the ongoing risk of deepening ethnic conflicts in Burma/Myanmar as the peace process is negotiated.

Burma/Myanmar is ranked 149 out of 168 countries on the Human Development Index (HDI). It has been characterized as a fragile state due to its governance record and ongoing conflict in many parts of the country (1). The successive military regimes that held power from 1962 to 2011 were engaged in ongoing conflicts with ethnic minority groups in many parts of the country. During that period, regime policies resulted in forced labor, forced relocation, torture, killings, and deliberate destruction of food supplies (2), largely targeted at ethnic minorities (3). The eastern Burmese border bore the largest burden of internally displaced people (IDPs) (4). During this time, there was no official government effort to provide health care for affected civilians in ethnic-controlled areas of the Karen/Kayin State (Fig. 1) (5).

**Fig. 1:**
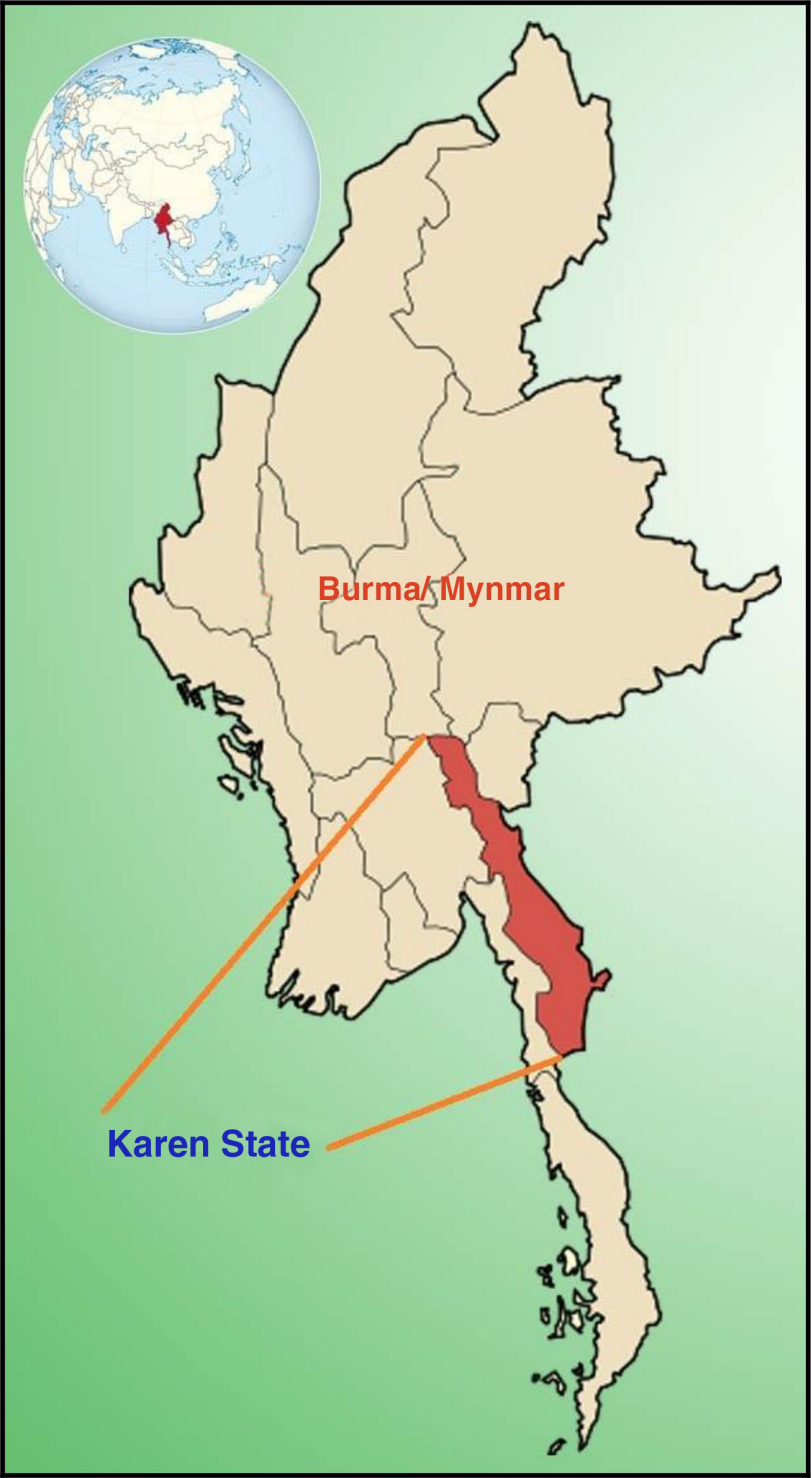
Location of Karen/Kayin State, eastern Burma/Myanmar.

Despite recent commitments to increase government spending on the health sector, the projections for health spending remains below 0.76% of gross domestic product (GDP) for 2012/2013 (6). Based on the 2009 World Health Organization (WHO) report, external donor support is also low (US$11.20 per capita) compared to regional counterparts like Cambodia and Laos, which are receiving US$22.20 and US$12.90 per capita, respectively (7). As a result, access to basic health services remains very poor in both ethnic-controlled and government-controlled areas of this region (3, 8, 9).

Due to the restricted national and international efforts (10, 11) to aid Burma/Myanmar’s IDPs, ethnic health organizations (EHOs) such as the Karen Department of Health and Welfare (KDHW) and community-based health organizations (CBHOs) such as the Burma Medical Association (BMA) and Back Pack Health Workers Team (BPHWT) were created to fill this gap. These entities play a crucial role in delivering essential health care to the IDPs in locations where, without these organizations, there would be none^1^ .

However, similar to many other challenging contexts, the health workers shortage is one of the main constraints of the Burma/Myanmar health system, as noted by Risso-Gill (2013). Nationally, the level of qualified human resources in Burma/Myanmar is an average of 1.4 doctors, nurses, and midwives (combined) per 1,000 persons (12), which is below the WHO critical shortage threshold of 1.7 health workers per 1,000 population^2^(13). This human resource shortage is even more amplified in the eastern ethnic-controlled border regions due to geographical remoteness, rural poverty, civil conflict, and limited access to education. As a result, *task shifting* was adopted as an innovative strategy to redistribute specialized primary health care (PHC) tasks that are usually administered by doctors and nurses to an active network of community-based medics, maternal health workers (MHWs), and community health workers (CHWs) (14, 15). More information about the tasks of each health worker to delivery PHC at village and village tract levels will be described in the ‘Findings’ section of this article.

The PHC service is operationalized collaboratively between the KDHW, BMA, and BPHWT in different townships and villages. The program routine data revealed that about 50% of the health services in the Karen State were delivered by the BPHWT mobile teams (Box 1). The other 50% were delivered by either KDHW and/or BMA clinics. Generally, the KDHW works in areas that are controlled by the Karen National Union (KNU), whereas the BMA and BPHWT are not tied to any political affiliation^3^ . For KNU areas, there are generally coordination and agreement between the three organizations, even though the target populations could overlap specifically for the coverage areas of BPHWT and KDHW mobile health teams. For the BPHWT teams, they would work only in areas where the community leaders see the need to be part of the BPHWT service coverage. Field staff from these organizations collect population data (Table 1) from their service area on an annual basis.

**Table 1:** Study area of ethnic townships: their names by government official and target population

No	Township	Government official name	Target population
1	Kawkareik	Kawkareik	45,356
2	Bu Tho	Hpapun	20,190
3	Lu Thaw	Hpapun	32,510
4	Na Bu	Myawaddy	34,697
5	Billin	Billin	23,494
6	Kyainseikgyi	Kyainseikgyi	14,440
7	Win Yee	Ye	30,516
8	Hlaing Bwe	Hlaingbwe	13,742
9	Htaw Ta Htoo	Htantabin	1,194
10	Dweh Loe	Hpapun	14,322
11	Kyauk Kyi	Kyaukkyi	6,792
12	K’ser Doh	Myeik	5,601
13	Shwe Kyin	Shwekyin	6,260
14	Ler Muh Lah	Myeik	4,614
Total target population			253,728

***Box 1*. EHOs and CBHOs in Karen/Kayin State**

The *Burma Medical Association* (BMA) was founded in 1991 by a group of health professionals from Burma/Myanmar. The BMA is an independent non-profit organization. Doctors, nurses, and other health professionals representing multiple ethnic groups gathered in Manerplaw, the former headquarters for the Karen National Union (KNU), to establish a forum for promoting health and human rights among displaced people from Burma/Myanmar. For the past 22 years, the BMA has been the leading body for health policy development and capacity building for the provision of quality health care services in ethnic areas of Burma/Myanmar.

The *Back Pack Health Worker Team* (BPHWT) was established in 1998 by Karenni, Mon, and Karen health workers to provide health care to internally displaced people living along the eastern border of Burma/Myanmar who have been affected by many decades of civil war. The BPHWT aims to improve health through the delivery of primary health care (PHC) and public health promotion. They provide medical care, community health education and prevention, maternal and child health care, and water and sanitation programs in the targeted field areas. Integrated through these PHC programs are health information and documentation and capacity-building programs.

In 1991, the KNU established the *Karen Department of Health and Welfare* (KDHW) to provide PHC to all people living in Karen/Kayin State. From 1991 to 1997, the KDHW administered the hospitals and clinics in all seven districts of Karen State, but the State Peace and Development Council (SPDC) offensive of 1997 decimated most of that health care infrastructure. In response, the KDHW organized the first mobile health clinic in 1998. Together with the Committee for Internally Displaced Karen People (CIDKP) and the BPHWT, the KDHW established additional mobile health clinics each year.

This article aims to advocate for the government and donors to acknowledge and build on the gains of the EHOs and CBHOs in health system strengthening, and not to risk alienating the local worker and ethnic communities. Although numerous studies had been conducted concerning health workers in eastern Burma/Myanmar on various topics, such as malaria knowledge (16), medic’s experiences of trauma and mental health (17), health workers’ strategies for addressing security and ensuring access to vulnerable ethnic communities (18), and perspectives from MHWs on delivering community-based care (19), this article is the first known attempt to document how the ethnic system of task shifting during the conflict period has effectively contributed toward the development of a strong ethnic health workforce to satisfy the essential health needs of the population in the EHOs’ and CBHOs’ service areas. It represents an empirical effort to map the health workforce (in their numbers, services, and training content) that is currently operating in Karen State.

## Data and methods

**Fig. 2:**
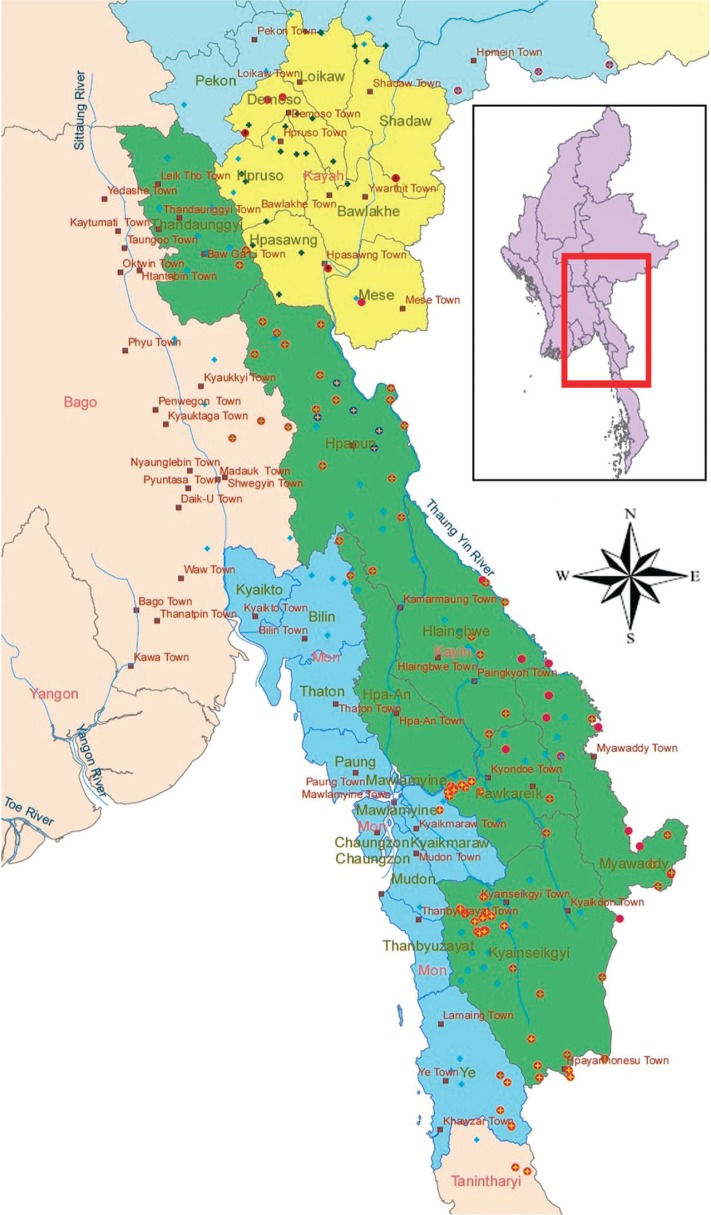
Study area of ethnic townships.

A case study approach was undertaken to document the structure of the health workforce in Karen State, and to compile comparable data related to the HRH, including their level of training and the health services that they delivered. This study is based on information collected through a rapid-mapping exercise that was conducted in 2012 with clinic-in-charges in 110 clinics in 14 Karen townships (Fig. 2). A one-paged survey questionnaire asking for information such as the number of health workers working in the clinics, their training, and the services provided by the clinics was used for the rapid mapping exercise.

Additional information on various levels of training and their outlines was obtained through a desk review of seven main organizational policy and training documents, which reflected the development of the HRH and were easily accessible. Ten informal context interviews were conducted with clinic-in-charges during their biannual meeting in Mae Sot. Interviews were facilitated by three health information system (HIS) staff. The initial draft of this article was forwarded for review and feedback by the five EHO and CBHO leaders who were integrally involved in the HRH development through policy, planning, or training. Two of them provided substantial guidance for the direction of this article.

No formal approval was obtained from an Institutional Review Board (IRB) as none exists in Karen State. However, the mapping exercise were reviewed and approved by individual EHOs and CBHOs as well as district-in-charges prior to data collection. The leaders also gave specific permission to the Health Information Systems Working Group (HISWG)^4^ to analyze the data collected from the rapid mapping. Additionally, there is no sensitive or intrusive information related directly to the population. The mapping was focused on HRH, their training, and the types of services provided to the community.

The issues investigated included five categories of interest: 1) density of the HRH, 2) systematic and rigorous training, 3) PHC service delivery, 4) level of cooperation and systematic approaches in task shifting, and 5) perceptions of task shifting among HRH. These themes were developed on the basis of lessons from the international literature, as well as the areas in which the EHOs and CBHOs are ready to engage in dialogues with the Burmese government, to mainstream HRH training and service delivery.

## Findings

### Density of HRH

Despite the lack of qualified health workers like doctors and nurses, the EHOs and CBHOs have been serving the population at the border through a lower-level cadre of health workers like medics, MHWs, and CHWs. Table 2 presents the assessment data from the rapid-mapping exercise where HRH at the aggregate level in the Karen State suggests no critical shortage of health workers, with a regional average of 2.8 medics, MHW, and CHW (combined) per 1,000 population (Table 2).

**Table 2:** Basic health workers’ densities in target population in Karen/Kayin state (authors’ calculations)

		Health workforce				Density per 1,000 population			
			
	Target population	Medic	MHW	CHW	Combined	Medic	MHW	CHW	Combined
BMA	42,358	25	23	59	107	0.6	0.5	1.4	2.5
KDHW	112,521	108	73	254	435	1.0	0.6	2.3	3.9
BPHWT	98,849	181	–	–	181	1.8	–	–	1.8
Total (14 townships)	253,728	314	96	313	723	1.2	0.4	1.2	2.8

The clinics and mobile teams work together in the community, with the clinics providing secondary care, performing deliveries, and providing specialized care. From a provider’s perspective^5^, it is projected that every mobile team (BPHWT) has roughly 1.8 health workers for a population of about 1,000 people, whereas each clinic (BMA or KDHW) has about 2.5–3.9 health workers for a population of about 1,000 people (Table 2). Although this density per 1,000 population for eastern Burma/Myanmar is higher than the national average, it should be noted that there is more division of labor in task shifting due to the vertical programming pushed out by donors through pooled funding such as the Three Millennium Development Goals (3MDG) (20), as well as due to the geographical remoteness of some of these areas.

### Systematic and rigorous training

Another critical element in task shifting is to ensure that the cadres that will be taking on the tasks are appropriately trained. Each of these health workers has gone through a set of systematic training, including an annual refresher. Biannually, they are also updated on the most current internationally approved case definitions and treatment protocols (21).

The EHOs and CBHOs trained the first cohort of medics in 1979. The curriculum has been revised several times and is now a 10.5-month training program. To date, a total of 27 cohorts have graduated and are working in the communities. The CHW program was first initiated in 1981 and was based on the curriculum set by the WHO. The training consists of an initial 6 months of theory followed by a 3-month practicum. In response to a shortage of skilled birth attendants in the Karen State, the EHOs and CBHOs established an 8-month MHW program, recruiting and training local women to deliver regular to complex care. This is to complement the pool of trained traditional birth attendants (TTBAs) and traditional birth attendants (TBAs) who have undergone 6- and 1-week trainings, respectively, with the EHOs/CBHOs.

International non-governmental organizations (INGOs) like the International Rescue Committee (IRC) and Community Partners International (CPI) work with the EHOs and CBHOs to build capacities in the areas of health workers’ training, HIS, and public health. They also coordinate with other CBHOs and the Mae Tao Clinic (MTC) (22), a community hospital in western Thailand serving the Burmese migrant and internal displaced people, in managing outbreak of diseases and referral of patients, as well as for medical training. There are also a number of ongoing training programs focusing on specific need areas like malaria and maternal and child health (MCH).

### Primary health care service delivery

To establish a framework for a sustainable health system by strengthening the health service delivery approaches in current EHO and CBHO areas, the tasks of medics, MHWs, and CHWs are clearly articulated in the basic package of health services for the Karen State:

Medical care: Involves medics who diagnose and treat the six common illnesses of malaria, acute respiratory infection (ARI), anemia, worm infestation, diarrhea, and dysentery. The medics are equipped with rapid diagnostic tests for malaria, as well as antimalarials, antibiotics for pneumonia and dysentery, and a variety of other essential medicines. They are trained in emergency care for injuries, and they have some referral sources for more complicated diseases.

MCH: MHWs are trained to use clean delivery kits, provide antenatal care (ANC) and postnatal care (PNC) to encourage safe deliveries and healthy infants, provide delivery and postpartum care, as well as distribute family planning (FP) methods. They are also trained in providing emergency obstetric care (EmOC), immunizations, and management of child health.

Community health education and prevention: Besides supporting the medics in diagnosing and treating the six common diseases, CHWs will also coordinate school health promotion activities, train village health workers (VHWs), and hold community health education workshops. Prevention activities include biannual deworming and vitamin A for children, as well as coordinating with village heads regarding the construction and maintenance of water systems and latrines.

### Level of cooperation and systematic approach to task shifting

In terms of organization, as mentioned in this article, the EHOs and CBHOs collaborate closely with each other and share responsibilities in a consensus-seeking manner.

The KDHW accordingly organizes coordination meetings every six month, in conjunction with the regular KDHW program meetings, field workshops, field operational meetings, and village workshops. – KDHW health worker

The clinic-in-charges from 15 field areas organized field meetings every 6 months, which included coordinated activities with ethnic health departments,local community based organizations, school teachers and leaders. – BMA health worker

**Fig. 3:**
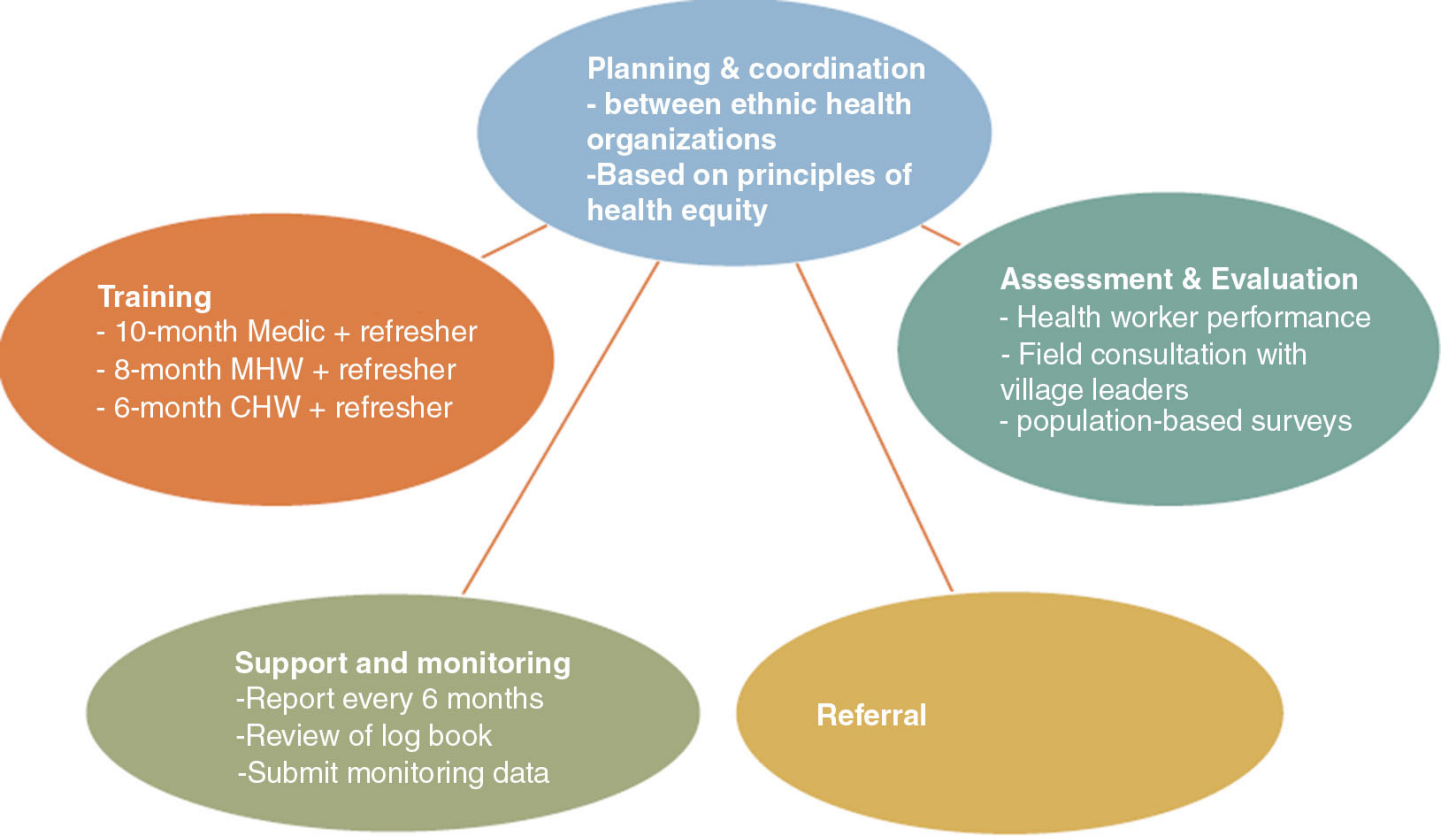
Systems approach in task shifting by EHOs.

From the informal interviews and protocols reviewed, it was apparent that EHOs and CBHOs have adopted a systematic approach in task shifting with the local delivery of health services (Fig. 3):

1. Planning and coordination between EHOs and CBHOs based on principles of health equity;
2. Training of medics (10 months), MHWs (8 months), and CHWs (6 months);
3. Support and monitoring once every 6 months with a review of log records and data;
4. Referral to high-level care; as well as
5. Assessment and evaluation of health workers’ performance, field consultation, and population-based surveys.

### Perception of task shifting among health workers

Health workers were generally positive about task shifting and the participatory system, which are necessary to succeed in the eastern Burma/Myanmar context and to ensure equity in access to health care with an emphasis on primary care and community self-reliance.

It depends on local people and local organizations. If they say their place is safe, we go there. We build makeshift tents with banana leaves and tarpaulin, and we give medical treatment. We can give medical treatment in some villages where the ethnic armed groups are in control. – BPHWT health worker

The incidence of diarrhea and cholera has decreased. Local community organizations have greater trust in our team … our approach is to go straight to the people and not to wait for the people to come to us. – BMA/BPHWT board member

## Discussion

Since voluntarily transitioning from a strict military regime to a quasi-civil government in the 2010 general election, Burma/Myanmar has undergone major political changes, indicating strong signals of willingness to reengage with the international community. However, decades of underinvestment and neglect of public services have resulted in a fragile and weak health system, which is reflected in poor health outcomes (23, 24). Whereas other parts of Burma/Myanmar may see improvements in development and access to health services as the economic and political framework of the country is reordered, it is likely that IDP communities in the Karen State will continue to be excluded from mainstream health services and may suffer further conflict as a result of the nation’s overall transition (5). A different strategy is needed for eastern Burma/Myanmar. This article outlines the ethnic service delivery model together with the strengths of its HRH, who are accepted by the population in this region.

### Task shifting ensures equitable access – health for all

The availability and accessibility of health facilities, goods, and services are critical under the Alma Ata right-to-health framework (25). Given the adequate health worker density and the systematic setup of training and refresher courses for the health workers in Karen State, task shifting has not demonstrated a detrimental effect on quality (26). Although this article did not seek to establish the direct effect of task shifting on population health status, it does show that a large number of people are receiving some level of vital health care in places where, otherwise, there would be none. This is coupled with the fact that EHOs and CBHOs generally administer clinical protocols that are recognized as effective at reducing morbidity and mortality in similar settings worldwide (27–31). For example, a relative comparative of proxy indicators from the household surveys conducted in 2008 and 2013 in the eastern Burma/Myanmar region showed indicative improvements in the population health status. For example, only 20% of children received vitamin A pills in 2008, but the percentage increased to 58.2% in 2013. Also, the prevalence of *Plasmodium falciparum* (PF) malaria was reduced from 6.9% in 2008 to 2.2% in 2013 (32, 33). This information clearly reflects the quality and importance of the health services provided by the existing health workforce. In the short and mid-run, task shifting increases the density of HRH reaching out to the underserved and hard-to-reach population.

### Task shifting and its relation to national HRH

Task shifting could be positioned as the entry point for policy engagement with the government. Task shifting to lower cadres of health workers among the EHOs and CBHOs only pertains to PHC provision. As Burma/Myanmar moves forward, PHC coverage will not be sufficient. Any arrangements to refer patients to either ethnic or national health systems will need to be tracked so that higher-level systems bottlenecks could also be addressed. If this issue of inadequate qualified health workers is not addressed, it could potentially reverse the success that task shifting has had to date.

In the long run, there is likely to be an increased demand for qualified health workers for the following reasons: 1) with a broader outreach to underserved and remote populations, health workers would connect those who are otherwise limited in access to qualified health workers; and 2) early detection of medical problems in underserved populations will thereafter increase demand on the formal health care system in the long run.

The EHOs and CBHOs are ready to discuss these topics with the Burmese government with the aim to improve national health system capacity, to promote investments across multiple disease areas and catalyze global improvements in health and survival, and to ensure that the ethnic populations that have not been served by the national health system will not be left behind.

### Recognition of parallel health systems

To support the health system strengthening of the Karen State, it is acknowledged that improved coordination among different EHOs and CBHOs is needed to reduce funding fragmentation. However, current challenges are the top-down and central-focus framework for implementing reform structures in the Burma/Myanmar health sector, with initiatives from the government to work with INGOs, EHOs, and CBHOs. By not acknowledging and building on the gains of the EHOs and CBHOs in health system strengthening, and by failing to acknowledge the meeting of health care needs through devolutive and distributive measures such as task shifting, top-down efforts risk duplicating services, engaging in inefficient funds distribution, and, most of all, alienating the local worker and ethnic communities.

This article broadly described the decentralized, community-based PHC system managed by EHOs and CBHOs with some secondary care offered by clinics. The referral mechanism is likely to be the weakest link between the parallel systems of the ethnic and government authorities (see Fig. 3). Pragmatic engagement between the two systems will extend the health care services closer to the communities where they are needed. In the long run, this engagement reduces costs on the national health system, and it ensures equity in access to health care with emphases on primary care and community reliance.

### Health systems reform in Burma/Myanmar and its relation to ASEAN integration

With the ongoing discussions on Association of Southeast Asian Nations (ASEAN) integration relating to migration and human mobility, it is crucial for the government, EHOs, and CBHOs to adapt their service delivery models to remain relevant. It is noteworthy to mention that for populations living in rural or remote areas with insufficient density of health workers, those who are closer to the borders will be likely to be accessing the providers across the Thailand border. Such diversion of care may increase inequities as those who lack resources often cannot afford travel costs.

A one-size-fits-all strategy and action plan will not work in the complex political and social contexts of the ASEAN region (34). In countries like the Philippines (35, 36) and Indonesia (37), whose governments have supported decentralization and the devolution of health services to rural and ethnic areas since 1991 and 2001, respectively, capacity and coordination challenges were reported with limited improved health outcomes. In Cambodia, however, the effectiveness of contracting non-state actors (NSAs) to deliver and manage services has been hampered by the widespread lack of transparency, the government’s failure to negotiate contracts openly, and the tendency of government officials to bypass laws and administrative processes in awarding contracts (38).

In the context of Burma/Myanmar, inequities in the coverage of health services are paralleled by similar disparities in the distribution of human and physical resources. Batley and Mcloughlin (39) mentioned that the most acute constraint on service provision specifically in conflict and postconflict situations is their dependence on not only the government’s capacity but also the capacity and willingness of NSAs, which will influence the potential for successful engagement.

As ASEAN integration gathers pace (40), whether the ethnic health systems will receive due recognition and practicing privileges is yet to be known. This article highlights the size, formality, and level of organization of the HRH in eastern Burma/Myanmar, and the EHOs’ and CBHOs’ willingness to engage with the government. This article attempts to serve as an important starting point for designing mutually beneficial forms of engagement in the area of HRH.

### Limitations and strengths of the study

We encountered a number of data limitations. First, although definitions and levels of training were similar across the organizations, how health workers operate in the field varies slightly. Second, organizations also adapted their service delivery models and medicine lists according to the funding requirements set by the donors. Finally, we had chosen to focus on analysis of data from HISWG and of data that are available based on the EHOs’ and CBHOs’ knowledge and experience of deploying health workers and providing services in conflict and postconflict periods.

This case study contributes to the limited literature on the role of task shifting in transitional and postconflict contexts like eastern Burma/Myanmar, where NSAs sought to be mainstream players in health system strengthening as well as service provision and management (39). This article does not seek to compare its findings with the health workforce of the government.

## Conclusion

Burma/Myanmar today is undergoing an unprecedented path of political and economic reforms. Improving access to and quality of care are actions of high priority among EHOs and CBHOs. Despite a landscape of chronic conflict until late 2010, this report provides evidence of the substantive system of health care provision and access over the years in eastern Burma/Myanmar. The constraints on funds flow and the level of engagement by external actors in this area during the last few years are imposing limitations on the delivery of basic services to the population in this region. This article underscores the climate of vulnerability of the EHOs and CBHOs due to a lack of regional and international understanding of this area’s political complexities as the media spotlight shines on the country’s economic and political reordering. Funding is highly fragmented with project-based services and programs, as challenges are typically addressed in a silo manner. The health workers are able to ensure basic health care to already vulnerable populations, but at the core of this has been a risk of loss of support in providing access to care.

## Footnotes

1 Burma/Myanmar is divided into zones, black zones, where free fire is allowed and the townships or villages are under ethnic administration; brown zones, where resistance groups are under control and the governance is of mix (ethnic and government) administration; and white zones where there is no fighting which means it is under government administration.

## Footnotes

2 The authors noted that WHO ratio of health care workers to population does not necessarily translate into “adequate” HRH. This paper does not set out to formally define the criteria that ensure ‘adequate’ services, e.g. is the mean a sufficient parameter to describe access to care?

## Footnotes

3 Other political organizations operating in the Karen/Kayin State could be the Democratic Karen Benevolent Army (DKBA) [formerly known as Democratic Karen Buddhist Army] and Border Guard Force (BGF). It should also be noted that both BMA and BPHWT operate beyond Karen/Kayin State, specifically BPHWT which also operates in Karenni, Mon States; Kayan, Shan Palaung, Pa-O and Lahu regions; as well as most recently Kachin and Arakan (Rakhine) states.

## Footnotes

4 The HISWG is a collaboration of seven (7) multi-ethnic EHOs and CBHOs as members [Back Pack Health Worker Team (BPHWT), Burma Medical Association (BMA), Karen Department of Health and welfare (KDHW), Karenni Mobile Health Committee (KnMHC), Mae Tao Clinic (MTC), Mon National Health Committee (MNHC) and Shan State Development Foundation (SSDF)], working together since 2002 to strengthen the health system for Eastern Burma/Myanmar, specifically through information/data sharing and management.

## Footnotes

5 It is noted that from the patient/villager perspectives, medical care might not be always available.
